# Psychoactive Substance Use and Associated Factors Among Adolescent Commercial Tricycle Riders in Tamale, Ghana: A Cross‐Sectional Study

**DOI:** 10.1002/hsr2.72787

**Published:** 2026-08-09

**Authors:** Yaa Nyarko Adjeso, Zaratu Bimonka Alhassan, Joseph Lasong

**Affiliations:** ^1^ Department of Population and Reproductive Health, School of Public Health University for Development Studies Tamale Ghana

**Keywords:** adolescents, prevalence, psychoactive substance, tricycle operators

## Abstract

**Background:**

Psychoactive substance use (PSU) has become a major public health concern. Many countries are alarmed by the increasing use of psychoactive substances among adolescents, and Ghana is not an exception.

**Aim:**

The study assessed psychoactive substance use among adolescent commercial tricycle riders of Tamale, Northern Region, Ghana.

**Methodology:**

A community‐based cross‐sectional study was conducted among 342 adolescent commercial tricycle operators selected from five (5) selected operational areas. Respondents were selected using a multistage sampling approach with simple random sampling within the operational areas, and a structured questionnaire was used as a data collection tool.

**Results:**

The prevalence rate of psychoactive substance use was 60.8%. Reasons for use include acceptability (20.4%), increased self‐confidence (60.8%), and improved work performance (24.6%). Factors that influenced psychoactive substance use include religious affiliation (Christian [aOR = 2.77 (CI: 1.44, 5.50), *p* = 0.003] or a Traditionalist [aOR = 4.76 (CI: 1.39, 20.75), *p* = 0.021]), fathers' educational level (aOR = 4.34 [CI: 1.38, 15.09], *p* = 0.015), large families (aOR = 2.55 [CI: 1.40, 4.79], *p* = 0.003), and peer and colleague introduction of psychoactive substances (aOR = 12.22 [CI: 3.16–63.48], *p* = 0.001).

**Conclusion:**

This study has shown a high level of psychoactive substance use among adolescent commercial tricycle operators within the Tamale metropolis, occurring within a context of poverty, hazardous child labor, and weak enforcement of child protection laws. Policy actions should include strict enforcement of the Children's Act, regulation of adolescent involvement in commercial transport, and coordinated interventions involving the Department of Children, Ghana Education Service, and National Road Safety Authority.

**Abbreviations**
LSD: lysergic acid diethylamide
OR: odds ratio
SDG: sustainable development goal
UNODC: United Nations Office on Drugs and Crime
WHO: World Health Organization

## 1 Background

According to Sustainable Development Goal (SDG) 3 target 3.5, understanding the prevalence and factors that contribute to psychoactive substance use (PSU) among adolescents is essential for the prevention and treatment of substance abuse [1, 2]. Studies have reported PSU among adolescents in sub‐Saharan Africa (SSA) [3, 4, 5, 6]. According to the World Health Organization (WHO), psychoactive substances are substances that, when taken in or administered into one's system, affect mental processes [7].

The reasons for the consumption of PSU range from increased work performance, increased sexual performance, and suppression of anxiety [8]. According to the United Nations Office on Drugs and Crime (UNODC), more than a quarter of a billion people worldwide use drugs. In 2018, an estimated 269 million people worldwide had used drugs at least once in the previous year (range: 166–373 million). This corresponds to 5.4% of the global population aged 15–64 (3.3%–7.5%), representing nearly 1 in every 19 people (UNODC World Drug Report 2020, 2020). This makes it a major public health challenge of global dimensions, as it results in physical, social, and psychological problems. Furthermore, a report from the WHO highlights that 0.6 million deaths per year are attributable to PSU, and notably, 0.4 million of the drug‐attributable deaths were among men [1].

Commercial tricycle use *(yellow‐yellow)* has quickly become the most convenient and preferred mode of transportation for most people in developing nations, including Ghana, despite the risk involved [9]. It also serves as a source of income for the growing number of unemployed youths [10]. Most of these tricyclists are youths, among whom a larger number are adolescents aged 15–19 years. In Tamale, especially, it has almost become the only mode of commercial transportation within the metropolis, with most of the operators not exempt from psychoactive substance use, which could pose a significant danger to themselves, their clients, and other road users. Adolescence is a developmental phase associated with a greater risk of experimenting and using substances such as tramadol, cannabis, and tobacco [11]. Studies have reported a higher prevalence of PSU among adolescents, and its use is associated with short‐ and long‐term effects on their health and safety as well as broader negative social consequences [11, 12, 13]. Notably, high prevalence rates of tobacco use (45.6%) are greater than those found in other studies around the world [14, 15, 16]. Further research found that cannabis use (7.09%) was most prevalent in West and Central Africa [17]. Estimates of cocaine use (4.16%) are greater than the 0.69% indicated in the World Drug Report 2015 [18]. Experimental curiosity, peer group influence, lack of parental supervision, personality difficulties owing to socio‐economic conditions, the need for energy for a long period, availability of drugs, and the need to avoid withdrawal disorder are among the reasons for PSU [17].

The use of psychoactive substances is prevalent among commercial tricycle operators (43.2%) [18] and commercial drivers and assistants (24.9%) [19] in Nigeria and Ghana, respectively. Half of the drivers who abused tramadol needed the drug to be able to carry out their daily activities effectively. Substance use is linked to a heightened risk of road traffic accidents, violence, sexual risk‐taking behaviors, mental health disorders, and suicide. Data from 2019, published by the Building and Road Research Institute (BRRI), indicate a fatality rate of 8.15 per 10,000 vehicles, with 829 crashes involving motorcycles, tricycles, and rickshaws. Additionally, the rate for motorcycle fatalities stands at 2.7 per 10,000 vehicles [20].

Although research has been conducted on substance use among adolescents in Ghana and tricycle riders in Nigeria, there is a lack of published studies specifically addressing substance use among adolescent tricycle riders. This study assessed the prevalence and factors associated with psychoactive substance use among adolescent tricycle riders in Tamale, Ghana.

## 2 Materials and Methods

### 2.1 Study Setting

The study setting is in the Tamale metropolis, the Northern Region of Ghana. It is one of the cities in Ghana and is rated as the fastest‐growing city in Ghana and West Africa as a whole, according to the Tamale Metropolitan Assembly. Tamale consists of 14 districts and is located 600 km north of Accra. It serves as the hub for all administrative and commercial activity in the Northern region, and its strategic location makes it the region's political, financial, and economic capital [21]. The tricycle has emerged as the most popular mode of transportation in Tamale, which causes many people to swarm to the area for their daily activities [9, 10, 20].

### 2.2 Study Population

The study population consisted of adolescent commercial tricycle operators between 10‐ and 19‐years old operating within the Tamale metropolis.

### 2.3 Study Design

A community‐based cross‐sectional study was conducted within the Tamale Metropolis.

### 2.4 Sample Size and Sampling

A minimum sample size of 311.2 was estimated using the Cochrane formula, *n* = (*Z* ^2^ *pq*)/*d* ^2^, where *n* is sample size, *Z* is the *z*‐score that corresponds with the 95% confidence interval (CI; 1.96), *p* is the estimated proportion of psychoactive substance use from a similar study with a prevalence of 28.2% [22], (*q* = 1 − *p* (1 – 0.282 = 0.718), and *d* is the margin of error, set at 5% (0.05). Assuming a non‐response rate of 10%, a sample of 342 was deemed appropriate to estimate the prevalence of psychoactive substance abuse and associated factors among adolescent tricycle riders in Tamale, Northern Ghana. However, complete data were available for 334 respondents, representing a response rate of 97.7%.

Multistage sampling was used in the selection of the respondents. In the first stage, five (5) operational areas (Aboabo market, Central taxi rank, Sakasaka, Lamehegu market, and Access bank area) for commercial tricycle activities in the Tamale metropolis were selected. These operational areas were purposively selected because of their high concentration of commercial tricycle activities and geographical distribution across the Tamale metropolis. In the second stage, a sampling frame was constructed by preparing a list of all adolescents of the target population in the five operational areas with the assistance of the station managers. Eligible adolescent commercial riders (10–19 years) who had been actively engaged in tricycle operations for at least 3 months and provided assent and consent from a legal guardian were allowed to participate in the study.

Within each operational area, simple random sampling was used to select respondents proportionate to the number of riders in each operational area. Due to the informal nature of this occupation, the sampling frame was based on locally compiled registers, which might not fully capture all eligible individuals; this limitation is acknowledged.

### 2.5 Data Collection Tool and Technique

The Drug Abuse Screening Test (DAST‐20), a validated drug questionnaire, was utilized to assess the prevalence of drug use. The DAST‐20 is a widely used instrument with established reliability and validity for identifying problematic drug use in clinical and community populations [23, 24, 25]. Although an adolescent‐specific version (DAST‐A) exists, the DAST‐20 has been applied in diverse populations, including young and non‐clinical groups, and allows comparability across studies. Its brevity, ease of administration, and prior use in community‐based substance use research informed its selection for this study. Furthermore, evidence from psychometric reviews supports its internal consistency and screening utility across heterogeneous populations [25]. However, we acknowledge that the use of DAST‐A may provide greater age‐specific sensitivity, which should be considered in future studies.

This questionnaire, which consisted of 20 [19] questions, was designed to gather information regarding potential involvement with drugs that did not involve alcoholic beverages over the previous year. Each question requires the respondents to indicate “yes” or “no.” A score of 1 is given for a Yes response except for items 4, 5, and 7, for which a No response is given a score of 1. The total summation is taken to a maximum score of 20.

The questionnaire was first translated into Dagbanli, Tamale, and then back into English by specialists working in the communication department of the University for Development Studies to ensure linguistic accuracy. To collect data, structured questionnaires were coded in the software application ODK (Kobo Collect), which served as the mechanism for data collection. Four research assistants received training on the content and utilization of Kobo Collect for data collection. A comprehensive collection of data was obtained on the sociodemographic characteristics and patterns of PSU, along with the specific types of substances used. We collected data over 3 months, from February 1, 2023 to April 30, 2023. Each participant used a maximum of 20 min to complete the data collection task.

### 2.6 Quality Assurance

The questionnaire underwent pretesting with 20 [19] tricycle operators in Savulugu and enabled the identification of regional terminologies for different substances, leading to minor revisions for clarity and contextual relevance. Participant data was secured and accessible only to the principal investigator.

### 2.7 Dependent Variable

Psychoactive substance use.

### 2.8 Independent Variables

Socio‐demographic features.

### 2.9 Data Analysis

The data were entered into the Statistical Package for Social Science (SPSS) version 25.0. Descriptive and inferential statistics were used for the analyses, and graphs and tables were used to present the findings. Bivariate analysis using the *χ* ^2^ test was used to assess the association between sociodemographic variables and psychoactive substance use. Variables with a *p* value of < 0.05 were included in the multivariable logistic regression model. Statistical significance was set at *p* < 0.05. Multicollinearity was assessed using variance inflation factors, and model fit was evaluated using the Hosmer‐Lemeshow goodness‐of‐fit test.

Continuous and categorical explanatory variables such as number of siblings, hours of riding per day, and years of riding experience were categorized to facilitate interpretation. Cut‐off points were determined based on the distribution of the data, guided by contextual relevance. The number of siblings was categorized as ≤ 5 and > 5 to reflect smaller versus larger family sizes. Daily riding hours were categorized as ≤ 10 and > 10 h to distinguish between standard and extended working durations, given that prolonged working hours are associated with fatigue and increased risk of behaviors among commercial drivers. Years of riding experience were categorized as ≤ 1 and > 1 to differentiate between relatively new riders and more experienced riders. Sensitivity to these categorizations is acknowledged, and these thresholds were primarily intended to enhance interpretability rather than imply clinically defined cutoff points.

### 2.10 Ethical Considerations

Ethical clearance was obtained from Kwame Nkrumah University of Science and Technology (CHRPE/AP/311/22). Administrative clearance was obtained from the station managers. Furthermore, respondents were taken through the purpose of the study, and written and thumb‐printed consent was obtained before the questionnaires were administered. Written parental consent was obtained from a legal guardian, either the mother, the father, or the guardian of the adolescent. Assent was obtained from all adolescents as well, after the study's rationale, benefits, potential risks, and assurance of confidentiality. Issues of confidentiality and anonymity were ensured. Respondents who needed support were referred to the appropriate unit as a means for risk mitigation. The Helsinki Declaration on Ethical Principles for Medical Research Involving Human Subjects, specifically, declarations 1–32 [26], was adhered to strictly during the study.

## 3 Results

### 3.1 Socio‐Demographic Characteristics of Respondents

Out of the 342 respondents, complete data were available for 334 respondents, representing a response rate of 97.7%. The majority were between the ages of 15 and 19 years (88.0%), Muslims (55.7%), and had completed junior high school (86.8%). The majority (69.5%) of the respondents had been driving commercial tricycles for more than a year (Table 1).

**Table 1:** Socio‐demographic characteristics of respondents.

Characteristics	Number (*n* = 334)	Percentage (%)
Age		
10– ≤ 14 years	40	12.0
15–19 years	294	88.0
Education level (respondent)		
None	12	3.6
Junior high school (JHS)^a^	290	86.8
Senior high school (SHS)^b^	32	9.6
Family size		
≤ 10	232	69.5
> 10	102	30.5
Religious affiliation		
Christianity	114	34.1
Muslim	186	55.7
Traditionalist	34	10.2
Average monthly income		
≤ 350cedis	188	56.3
> 350cedis	146	43.7
Father's education level		
None	43	12.9
Basic/higher education	160	47.9
Tertiary education	131	39.2
Mother's education level		
None	122	36.5
Basic/higher education	183	54.8
Tertiary education	29	8.7
Tricycle ownership		
Family owned	107	32.0
Not family‐owned	227	68.0
Years of riding tricycle		
≤ 1 year	102	30.5
> 1 year	232	69.5
Ever had an accident during riding		
No	169	50.6
Yes	165	49.4
Adherence to traffic regulations		
No	149	44.6
Yes	185	55.4
Hours of riding		
≤ 10 h	114	34.1
> 10 h	220	65.9
Reasons for riding		
Pleasure	19	5.7
For income	315	94.3

^a^ Junior high school (basic): 9 years of formal school, 6 years of primary education, and 3 years of junior high school).

^b^ Senior high school (higher): 3 years of secondary education.

### 3.2 Prevalence and Self‐Reported Commonly Used Substances Among Respondents

**Figure 1:**
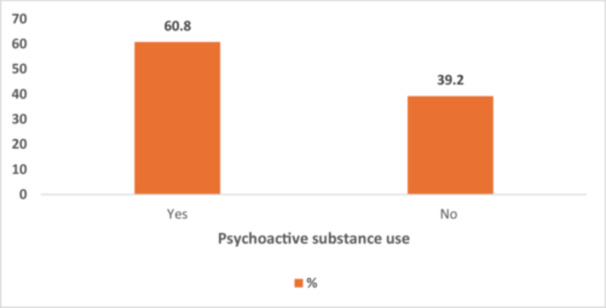
Prevalence of psychoactive substance use among respondents.

**Figure 2:**
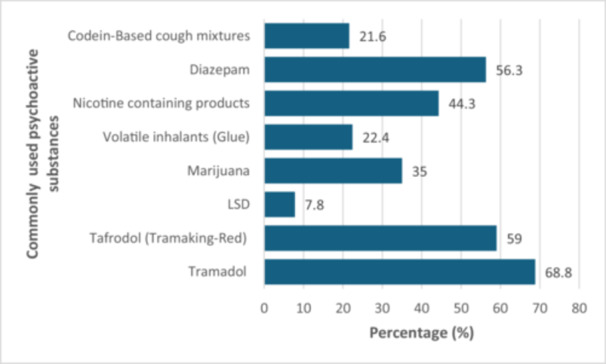
Self‐reported use of common psychoactive substances by adolescent commercial tricycle riders. LSD, lysergic acid diethylamide. Multiple responses were allowed.

Most adolescent tricycle riders (60.8%) use psychoactive substances (Figure 1). The commonly abused substances are Tramadol (68.8%), Trafodol (*wonim red*) (59.0%), diazepam (56.3%), marijuana (35.0%), nicotine‐containing substances (44.1%), and volatile inhalants (*superglue*) (22.4%) (Figure 2).

### 3.3 Reasons for Substance Use Among Respondents

**Figure 3:**
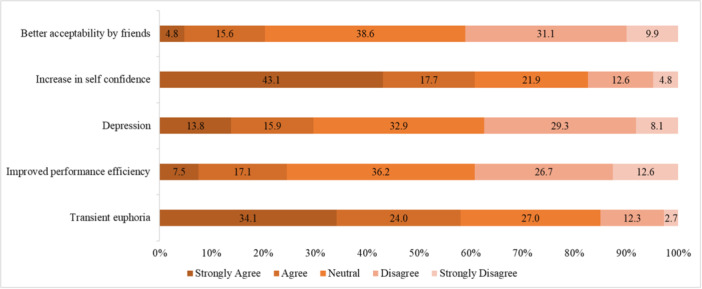
Respondents' reasons for using psychoactive substances.

The reasons for psychoactive substance use included better acceptability by friends (20.4%), self‐confidence (60.8%), and improved work performance (24.6%) (Figure 3).

### 3.4 Association Between Socio‐Demographics and Psychoactive Substance Use

In Table 2, Christians and Traditionalists were 1.91 and 5.32 times more likely to use psychoactive substances than those who were Muslims (cOR = 1.91 [CI: 1.17, 3.11], *p* = 0.009) and (cOR = 5.32 [CI: 1.97, 14.35], *p* = 0.001), respectively. Respondents whose fathers had basic education were 3.90 times more likely to use psychoactive substances than those with advanced (tertiary) education (cOR = 3.90 [CI: 2.37, 6.43], *p* < 0.001). Respondents whose fathers were informally employed were 3.2 times more likely to use psychoactive substances than those with formal employment (cOR = 3.2 (CI: 2.00, 5.33), *p* < 0.001). Respondents with more than 5 siblings were 3.13 times more likely to use psychoactive substances than those with less than 5 siblings (cOR = 3.13 [CI: 1.89, 5.19], *p* < 0.001). Respondents who have been riding tricycles for more than a year were 2.14 times more likely to use psychoactive substances than those riding for less than a year (cOR = 2.14 [CI: 1.33, 3.43], *p* = 0.002). Respondents who ride tricycles for more than 10 h were 39% less likely to use psychoactive substances than those who ride less than 10 h (cOR = 0.61 [CI: 0.38, 0.98], *p* = 0.040). Respondents who contributed money for housekeeping were 2.19 times more likely to use PS than those who did not contribute (cOR = 2.19 [CI: 1.36, 3.53], *p* = 0.001).

**Table 2:** Bivariate analysis of respondents' characteristics and psychoactive substance use.

	Psychoactive substance use			
Characteristics	No (*n* = 131)	Yes (*n* = 203)	*χ* ^2^ (*p* value)	cOR (C.I.) *p* value
Age group				
10– ≤ 14 years	12 (9.2)	28 (13.8)		1
15–19 years	119 (90.8)	175 (86.2)	1.62 (0.203)	0.63 (0.31, 1.29) 0.21
Respondents education				
None	5 (3.8)	7 (3.4)		
Junior High School (JHS)	110 (84.0)	180 (88.7)		1.17 (0.36, 3.77) 0.79
Senior High School (SHS)	16 (12.2)	16 (7.9)	1.79 (0.408)	0.71 (0.18, 2.72) 0.62
Family size				
≤ 10	98 (74.8)	134 (66.0)		1
> 10	33 (25.2)	69 (34.0)	2.90 (0.088)	1.53 (0.94, 2.50) 0.09
Religious affiliation				
Muslim	37 (28.2)	77 (37.9)		1
Christianity	89 (67.9)	97 (47.8)		1.91 (1.17, 3.11) 0.009
Traditionalist	5 (3.8)	29 (14.3)	16.56 (< 0.001)	5.32 (1.97, 14.35) 0.001
Income				
< 350cedis	70 (53.4)	118 (58.1)		1
> 350cedis	61 (46.6)	85 (41.9)	0.713 (0.399)	0.83 (0.53, 1.29) 0.39
Mother's education				
Tertiary education	61 (46.6)	122 (60.1)		1
Basic/higher education	14 (10.7)	15 (7.4)		1.87 (0.84, 4.12) 0.12
None	56 (42.7)	66 (32.5)	5.94 (0.051)	1.1 (0.48, 2.47) 0.82
Father's education				
Tertiary education	73 (55.7)	58 (28.6)		1
Basic/higher education	39 (29.8)	121 (59.6)		3.90 (2.37, 6.43) < 0.001
None	19 (14.5)	24 (11.8)	30.21 (< 0.001)	1.59 (0.79, 3.18) 0.19
Father's occupation				
Formal	74 (56.5)	62 (30.5)		1
Informal	42 (32.1)	115 (56.7)		3.2 (2.00, 5.33) < 0.001
Unemployed	15 (11.5)	26 (12.8)	23.52 (< 0.001)	2.06 (1.00, 4.24) 0.05
Mother's occupation				
Formal	18 (13.7)	17 (8.4)		1
Informal	80 (61.1)	142 (70.0)		1.88 (0.91, 3.85) 0.09
Unemployed	33 (25.2)	44 (21.7)	3.56 (0.169)	1.41 (0.63, 3.15) 0.34
Number siblings				
≤ 5	104 (79.4)	112 (55.2)		1
> 5	27 (20.6)	91 (44.8)	20.44 (< 0.001)	3.13 (1.89, 5.19) < 0.001
Reason for riding				
Pleasure	12 (9.2)	7 (3.4)		1
For income/support	119 (90.8)	196 (96.6)	4.84 (0.028)	2.82 (1.08, 7.37) 0.034
Ownership of tricycle				
Family owned	47 (35.9)	60 (29.6)		1
Not family‐owned	84 (64.1)	143 (70.4)	1.46 (0.227)	1.33 (0.84, 2.13) 0.23
Years of riding				
≤ 1 year	53 (40.5)	49 (24.1)		1
> 1 year	78 (59.5)	154 (75.9)	9.99 (0.002)	2.14 (1.33, 3.43) 0.002
Ever had an accident				
No	90 (68.7)	79 (38.9)		1
Yes	41 (31.3)	124 (61.1)	28.26 (< 0.001)	0.65 (0.41, 1.01) 0.06
Obey traffic regulation				
No	50 (38.2)	99 (48.8)		1
Yes	81 (61.8)	104 (51.2)	3.62 (0.057)	0.65 (0.41, 1.01) 0.06
Hours of riding				
≤ 10 h	36 (27.5)	78 (38.4)		1
> 10 h	95 (72.5)	125 (61.6)	4.24 (0.039)	0.61 (0.38, 0.98) 0.04
Drug introduction				
No‐one	29 (22.1)	3 (1.5)		1
Parent	3 (2.3)	9 (4.4)		29.00 (4.96, 169.65) < 0.001
Peers/colleagues	99 (75.6)	191 (94.1)	39.63 (< 0.001)	18.65 (5.54, 62.74) < 0.001
Contribution money				
No	53 (40.5)	48 (23.6)		1
Yes	78 (59.5)	155 (76.4)	10.67 (0.001)	2.19 (1.36, 3.53) 0.001
Ability to operate				
No	43 (32.8)	36 (17.7)		1
Yes	88 (67.2)	167 (82.3)	10.04 (0.002)	2.27 (1.36, 3.78) 0.002

*Note:* Pearson's *χ* ^2^ test, odds ratio (binary logistic regression), C.I. (confidence interval).

### 3.5 Predictors of Psychoactive Substances Use Among Respondents

**Figure 4:**
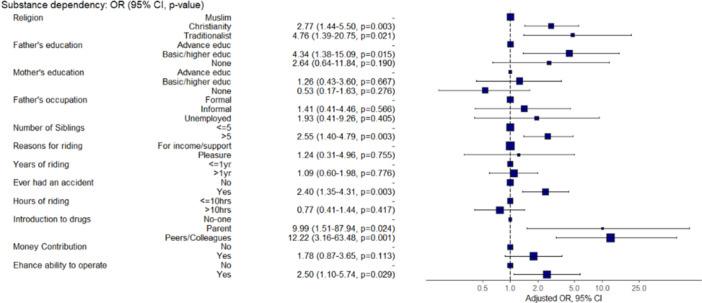
Factors associated with psychoactive substances use among respondents.

After adjusting for additional variables, respondents identifying as Christians (aOR = 2.77 (CI: 1.44, 5.50), *p*= 0.003) and traditionalists (aOR = 4.76 (CI: 1.39, 20.75), *p*= 0.021) showed a 2.77‐ and 4.76‐times greater likelihood of psychoactive substance use, respectively, compared to Muslims. Respondents whose fathers had basic education (aOR = 4.34 (CI: 1.38, 15.09), *p*= 0.015) were 4.34 times more likely to use psychoactive substances than those with advanced (tertiary) education. Respondents with more than 5 siblings were 2.55 times more likely to use psychoactive substances than those with less than five siblings (aOR = 2.55 (CI: 1.40, 4.79), *p*= 0.003). Respondents with a history of accidents were 2.40 times more likely to use psychoactive substances than those with no history (aOR = 2.40 (CI: 1.35, 4.31), *p*= 0.003) (Figure 4).

## 4 Discussion

This study found a high use of psychoactive substances (60.8%) among adolescent commercial tricycle riders in the Tamale metropolis. Substance use was associated with religious affiliation, father's education and occupation, family size, history of accident, and drug introduction by peers and parents. The most reported reasons for use were increased self‐confidence, improved work performance, and peer influence. These findings highlight the relationship between adolescent vulnerability, informal employment, and substance use.

Most of the commercial tricycle riders were older adolescents (15–19 years) (88.0%) [27], and 60.8% used psychoactive substances. Adolescents' usage of substances is also progressively becoming a bigger issue [28], especially in the Tamale metropolis. A study showed that most young people use drugs covertly to cope with anxiety, depression, eating disorders, and other behaviors that make them feel inadequate when compared to others [16]. Studies have shown that those who first started abusing psychoactive substances at a young age have the highest risk of being addicted [11, 12, 18, 19, 29, 30]. Again, when a person is young, it is a susceptible stage, and substance experimentation is pleasing [30]. The prevalence of psychoactive substance use among adolescent commercial tricycle riders within the Tamale metropolis was higher than the prevalence reported among commercial drivers and adolescents in other settings in Ghana and sub‐Saharan Africa (SSA). A study reported a prevalence of 12.3% among adolescents in Ghana [11] and a prevalence of 24.9% among commercial drivers and assistants [19] in the Greater Accra Region. In another study conducted in eight SSA countries, among school‐going adolescents (11–18 years), 11.3% used drugs; 2% used marijuana, and 2.6% had used amphetamines at some point in their lives [11]. The highest prevalence rate of psychoactive substance use was 66.5% among Ethiopian university students [3]. This difference might be explained by the unique characteristics of the study population, that is, adolescents engaged in informal and physically demanding work, often for extended working hours and under economic pressure. The notion that using drugs helps in working, which is common among adolescents, might be a factor in the higher incidence of psychoactive substance use [3, 21].

Further, the presence of commercial tricycle riders aged 10–14 years raises important legal and child protection concerns. Ghana ratified the United Nations Convention on the Rights of the Child in 1990 and enacted the Children's Act (1998), which sets the minimum working age at 15 and prohibits hazardous work for individuals under 18 years of age [31]. Commercial tricycle riding, involving long hours, traffic exposure, and increased risk of road traffic accidents, constitutes hazardous work. Consequently, the participation of young adolescents in this occupation, therefore, reflects not only a public health issue but also systemic failure in the enforcement of child protection laws.

Additionally, the pattern of psychoactive substances used ranged from opioids, depressants, and cannabis, which suggests that psychoactive substance use in this group might be functionally driven. Other studies show that adolescents in high schools commonly use tobacco (72.9%), marijuana (32.4%), valium (16.7%), and librium (7.1%) [32]. Respondents reported using substances to enhance work performance, reduce fatigue, and increase confidence. The disparity in psychoactive substance abuse might stem from variations in where the study was conducted and the availability of these substances [6]. This has highlighted the usefulness of law enforcement organizations in regulating psychoactive substances in these areas and the capacity of drug traffickers to surpass these organizations, thus ensuring the availability of psychoactive substances to users [33]. Other studies have reported similar findings among commercial drivers, where psychoactive substances are used to cope with long working hours and occupational stress [19]. This functional use raises important concerns regarding road safety, as substances may impair judgment [34], reaction time, and overall driving performance. It is essential to implement more rigorous mechanisms and measures to restrict access to these psychoactive substances and to ensure that identified users receive the necessary treatment tailored to their specific needs [19, 29].

Parental and peer/colleague influence appeared as important factors, with a substantial proportion of respondents reporting introduction to substances through peers and parents [34]. This finding aligns with existing evidence that peer networks play a critical role in shaping adolescent risk behaviors, particularly in informal environments where regulatory oversight is limited [19, 35]. Further, the extremely high odds associated with peer and colleague introduction highlight the occupational environment as a critical driver of substance use. Adolescents in this setting work alongside older riders for prolonged hours, where substance use may be normalized and socially reinforced. At a developmentally vulnerable stage, repeated exposure to older co‐workers who use psychoactive substances may facilitate initiation, imitation, and sustained use. This suggests that adults' co‐workers are not passive influences but active risk factors, indicating that interventions must extend beyond adolescents to include workplace‐based and peer network interventions [36] and behavior change communication strategies [19, 30].

The study also identified significant associations between socio‐demographic characteristics and psychoactive substance use. Adolescents from larger families were more likely to use substances than those from smaller families [36], which might reflect reduced parental supervision and increased economic pressures [37]. Similarly, lower levels of fathers' education were associated with higher odds of psychoactive substance use. The result supports findings from another study that suggested a connection between fathers' education and substance use [38]. Adolescents' psychoactive substance use has been linked to parental and other relatives' educational levels, as well as a variety of attitudes and beliefs, including participation in risky behaviors [39]. Beyond individual behavioral factors, the findings point to structural vulnerabilities influencing psychoactive substance use among adolescent riders. The convergence of low parental education, informal employment, large family size, and adolescents' financial contribution to household income reflects a structural pattern consistent with poverty‐driven child labor. These findings suggest that adolescents' involvement in commercial tricycle operations is not merely an economic activity but may represent a form of economic exploitation driven by household survival needs. These socioeconomic pressures may increase susceptibility to psychoactive substance use as adolescents attempt to cope with prolonged working hours, occupational stress, and financial responsibility. These findings highlight the importance of addressing the social determinants of health underlying adolescent substance use rather than focusing solely on individual‐level behavioral change.

Respondents with a history of accident were 3.13 times more likely to use substances (cOR = 3.13 [CI: 1.89, 5.19], *p* < 0.001) than those with no history of accident. The rate of vehicular accidents and associated injuries and deaths in Ghana has recently increased. Studies have established that some accidents are due to psychoactive substance use by drivers [33]. Driving under the influence of drugs could pose a threat not only to the driver but also to passengers and pedestrians.

Further, the association between psychoactive substance use and road traffic accidents reported in this study is particularly concerning. Adolescents who had ever had an accident were more likely to use substances, suggesting a potential bidirectional relationship. Studies have established that some road accidents are due to substance abuse by drivers, and involvement in accidents might also lead to substance use as a coping mechanism for injury or stress [39]. Although causality cannot be established due to the cross‐sectional design, this finding underscores the need for integrated road safety and substance use prevention strategies.

While substance use education remains important, behavioral interventions alone are unlikely to sufficiently address the structural and occupational conditions driving psychoactive substance use among adolescent riders. A multisectoral response involving public health institutions, child protection agencies, transport regulatory bodies, educational authorities, and local government structures is needed [40].

## 5 Limitation

The cross‐sectional design makes causal inference difficult to identify, and the use of self‐reported data might introduce recall and social desirability bias. Additionally, the categorization of certain variables, such as family size and working hours, was based on data distribution and contextual considerations rather than standardized thresholds, which might affect comparability with other studies. Again, the informal nature of the study population made it challenging to construct a comprehensive sampling frame. Station managers assisted with the development of the sampling frame, which might have introduced gatekeepers' bias. Managers could have excluded irregular or non‐affiliated riders, potentially affecting representativeness. A general population screening tool was used instead of an adolescent‐specific instrument (DAST‐A) in adolescent respondents. Despite these challenges, the study has several strengths. It focused on hard‐to‐reach and understudied populations, providing novel insights into psychoactive substance use among adolescent commercial tricycle riders in Ghana. The use of a standardized screening tool enhanced the reliability of the findings, and the relatively large sample size improved the robustness of the analysis.

## 6 Conclusion

The study found a high prevalence of psychoactive substance use among adolescent commercial tricycle riders of the Tamale metropolis, occurring within a context of poverty, hazardous child labor, and weak enforcement of child protection laws. The findings suggest gaps in the enforcement of child protection and transport regulations in Ghana. Addressing psychoactive substance use in this population requires a multisectoral strategy involving enforcement of the Children's Act, regulation of adolescents in commercial transport activities, peer and workplace‐based interventions, school retention initiatives, and targeted social protection measures for economically vulnerable households.

## Author Contributions

**Yaa Nyarko Adjeso:** conceptualization, methodology, supervision, writing – original draft, formal analysis, visualization, resources, data curation, and project administration. **Zaratu Bimonka Alhassan:** conceptualization, methodology, writing – review and editing, and resources. **Joseph Lasong:** conceptualization, writing – review and editing.

## Funding

The authors have nothing to report.

## Ethics Statement

Ethical clearance was obtained from the Kwame Nkrumah University of Science and Technology (CHRPE/AP/311/22). Administrative clearance was obtained from the station managers before conducting the study. Furthermore, participants were informed about the purpose of the study, and their consent was obtained before the questionnaires were administered. Issues of confidentiality and anonymity were ensured.

The study equally observed and adhered to the Helsinki Declaration on Ethical Principles for Medical Research Involving Human Subjects, specifically, declarations 1–32.

## Conflicts of Interest

The authors declare no conflicts of interest.

## Transparency Statement

The lead author, Yaa Nyarko Adjeso, affirms that this manuscript is an honest, accurate, and transparent account of the study being reported; that no important aspects of the study have been omitted; and that any discrepancies from the study as planned (and, if relevant, registered) have been explained.

## Data Availability

The datasets used and/or analyzed during the current study are available from the corresponding author on reasonable request.
